# Clinical evaluation of S1 alar screws application in short-segment lumbosacral fixation and fusion for spine infection with severe S1 vertebral body loss

**DOI:** 10.1186/s12891-022-05824-6

**Published:** 2022-09-16

**Authors:** Weizhi Fang, Weijun Liu, Qingbo Li, Lei Cai, Wei Wang, Xincheng Yi, Hongbo Jiao, Zhi Yao

**Affiliations:** 1 Department of Orthopaedics, Wuhan Fourth Hospital, No. 473 Hanzheng Street, Qiaokou District, Wuhan, China; 2 Wuhan Fourth Hospital; Puai Hospital, Tongji Medical College, Huazhong University of Science and Technology, No. 473 Hanzheng Street, Qiaokou District, Wuhan, China

**Keywords:** S1 alar screw, Spinal infection, Lumbosacral fixation, Short-segment

## Abstract

**Background:**

The one-stage posterior approach for treating spinal infection has recently been generally accepted. However, severe vertebral body loss caused by infection remains a major challenge in posterior surgery. This study was conducted to evaluate the clinical application and outcomes of S1 alar screws used in the one-stage posterior surgery of short-segment lumbosacral fixation and fusion after debridement for infection with severe S1 vertebral body loss.

**Methods:**

The clinical features and treatment outcomes of 7 patients with spinal infections from August 2016 to August 2021 who were treated with one-stage posterior surgery using S1 alar screws were retrospectively analyzed. The clinical data, including patient data, visual analogue scale (VAS), Oswestry Disability Index (ODI), fusion time and complications of the patients, were recorded.

**Results:**

All 7 patients were followed up for an average duration of 14.57 months (range, 12—18 months). The VAS score decreased significantly from 7.3 preoperatively (range, 6—8) to 2.6 postoperatively (range, 2—3). The ODI score demonstrated a steady and gradual increase from 73.8 preoperatively (range, 68—75) to 33.6 postoperatively (range, 30—37). Bony fusion time was observed approximately 6.8 months after surgery. Two patients in our study experienced the postoperative local pain, which could be relieved by analgesics and disappeared 3 months after the operation. There were no complications of intraoperative fracture, posterior wound infection or neurovascular injury.

**Conclusions:**

S1 alar screws are suitable for use in the operation and could be an alternative option to S1 pedicle screws for short-segment lumbosacral fixation and fusion with severe S1 vertebral body loss caused by spinal infection, which could provide satisfactory clinical outcomes.

## Background

Spine infection refers to an infection affecting the intervertebral disk, the vertebral body or the paravertebral structures; and the cause of disease can be mainly classified as postoperatively or natively pyogenic and tuberculosis [1]. The incidence of spinal infections has been reported to vary between 0.5% and 0.1% each year and is steadily rising [2]. Spine infections are rare but can be severe and life-threatening. The most frequently involved spinal segment is the lumbar spine (58%), and the most common symptoms reported are back pain (85%), followed by fever (48%) and paresis (32%) [3]. Surgical intervention for spine infection is recommended for patients with compromised neurological function and significant kyphotic deformity or instability [4], nevertheless the surgical approach is still a controversial issue whether internal fixation is required and whether anterior or posterior approach is performed [5]. The anterior approach is convenient for debriding infection and reconstructing stability but demands highly technical skills and a large skin incision during surgery, which can lead to trauma [6]. To avoid considerable surgical invasiveness and blood during surgery by means of the anterior approach, an increasing number of surgeons have recently adopted one-stage posterior instrumentation in the treatment of spine infection using pedicle screws [7], which are widely used in segments of the S1 vertebral body.

The posterior operation of internal fixation and fusion with or without debridement has proven to be safe and efficient in resolving spinal infection [8]. Posterior instrumentation for spinal infection could be used to apply sufficient compression forces on the anterior grafts, prevent slippage of the grafts and promote intervertebral fusion [9]. According to reports, posterior short-segment fixation and fusion surgery in lumbosacral tuberculosis is a safe and cost-effective means of treatment with satisfactory functional recovery [10]. However, for lumbosacral infections with severe S1 vertebral body loss, S1 pedicle screws cannot provide stable fixation. Moreover, screws oriented toward the infected vertebral body are unsuitable. In addition to S1 pedicle screws, many kinds of lumbosacral fixation techniques have been developed in recent decades [11]. Iliac screws and S2-alar-iliac (S2AI) screws are most widely used today and can achieve high rates of fusion and great biomechanical stability, but rates of reoperation, instrument failure, and surgical morbidity remain major challenges in sacropelvic fixation [12].

It is important to find an alternation for pedicle screws and other screws, such as iliac screws and S2AI screws, as internal fixation instruments in one-stage posterior short-segment fusion when the S1 vertebral body is infected or damaged due to lumbosacral infection. We suppose that S1 alar screws oriented to the usually uninfected lateral mass of the sacrum may be an alternative for pedicle screws. According to the report [13], the bone mineral density (BMD) of the first sacral segment was significantly higher than that of the second sacral segment. A cadaver study of 13 sacral specimens from young men showed that the BMD of the upper and lateral mass of the sacral ala was relatively higher than that of other areas of the sacral ala, except that of the pedicle area [14]. This research on the BMD characteristics of the first sacral segment led to the conclusion that S1 alar screws may have the potential to be suitable for short-segment lumbosacral fusion.

Therefore, we presume that for patients who suffer from lumbosacral infection with severe vertebral body loss, S1 alar screws may be an effective alternative to S1 pedicle screws for the treatment of short-segment fusion after debridement. To date, there are no clinical reports about the application of S1 alar screws in lumbosacral fusion. Meanwhile, to explore the feasibility of the S1 alar screws and offer guidance for screw entrance during an operation, a parallel study of the radiological characteristics of the optimal trajectory of the S1 alar screws was performed.

In this study, we evaluated the clinical application and outcomes as well as the radiological parameters of S1 alar screws used in one-stage posterior surgery of short-segment lumbosacral fixation and fusion after debridement for infection with severe S1 vertebral body loss to find more options for these patients.

## Methods

### Study design

We conducted a retrospective review of the clinical and radiological data obtained from August 2016 to August 2021 in Wuhan Forth Hospital. Seven patients underwent surgery within the short-segment lumbosacral fixation to fuse the low lumbar spine (L4 or L5) to the first sacral vertebra using bilateral S1 alar screws. The same senior surgeon performed surgery on all patients for different reasons, such as tuberculotic spondylitis, and postoperative or natively pyogenic spondylitis. The inclusion criteria for the present study were as follows: (1) one or two levels of low lumber spine fusion to the sacrum, including L5/S1 and/or L4/L5 and (2) a minimum follow-up of 12 months. Exclusion criteria were as follows: (1) patients who underwent fusion surgery ≥ 3 levels; (2) patients who were unsuitable for internal fixation due to severe sacrum damage or osteoporosis(BMD ≤ 2.5 SD)resulting from other diseases.

### Surgical procedure

**Fig. 1:**
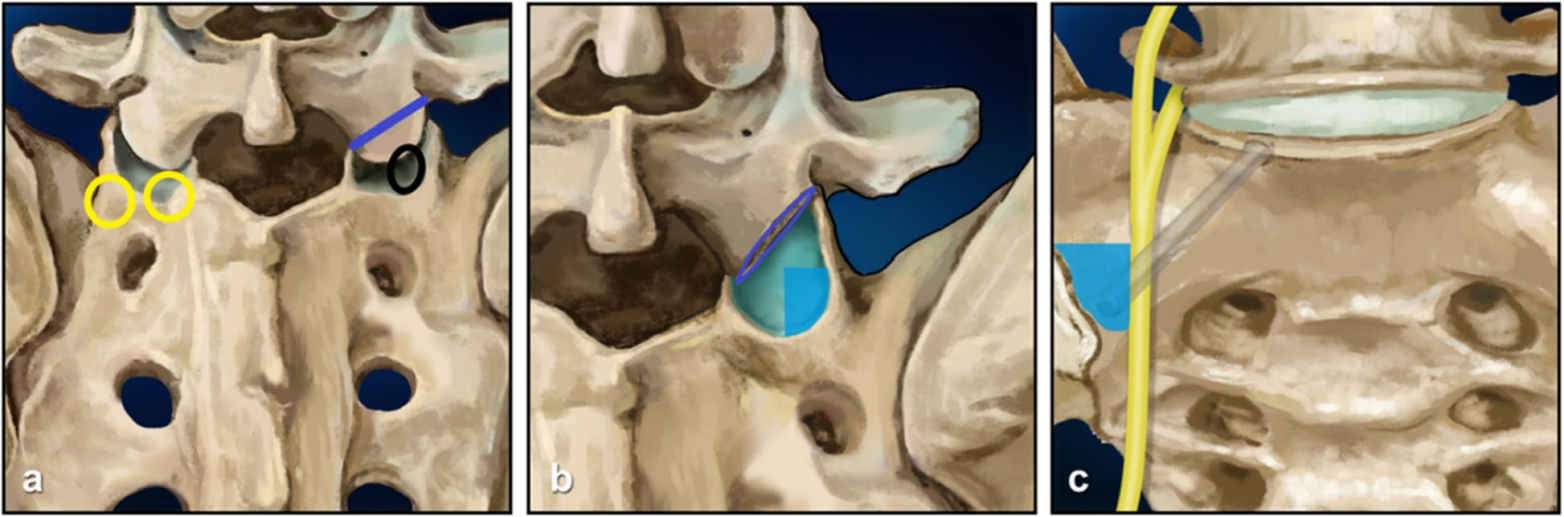
The unique entry point and the trajectory. **a** The entry points (left two yellow circles) of S1 screws described in the previous study were medial and inferior to the S1 facet (Mirkovic,1991) and 5 mm inferior and 10 mm lateral to the S1 facet (Asher,1986). The cutting line of osteotomy was at the L5 articular process to expose the inferior half of S1 superior articular process (right blue line and black circle). **b** The cross section of the L5 articular process inferior (cyan) and the unique entry point (blue area) at the lateral inferior part of the inferior half of S1 superior articular surface (blue area). **c** The trajectory of the S1 alar screw (magenta line) and the safe zone (blue area) on the lateral side of the lumbar 5 nerve root

Standard posterior laminectomy and internal screw fixation were performed in each patient under general anesthesia. The inferior L5 articular process was resected to expose the unique starting point of the S1 alar screws at the lateral inferior part of the inferior half of the S1 superior articular surface (Fig. 1). The spinous process and articular process of the L5 vertebrae were resected to create operating space for trajectory preparation and screw placement. An awl was used to create a tunnel directed 30° lateral and 45° inferior at a depth of approximately 40 mm into the lateral sacral ala according to preoperatively measured data. After conforming to the direction of the tunnel by intraoperative fluoroscopy and determining the integrity of the bone tunnel by a blunt probe, screws 7.0–7.5 mm in diameter and 35–45 mm in length were inserted into the same trajectory.

For structural bone grafting in all included patients, two blocky bones were obtained from the iliac bone and trimmed to a suitable size of 22–26 mm in length, 10–12 mm in width and 12–16 mm in height. After appropriate distraction of the intervertebral space with the posterior instruments and thorough debridement, the blocky bones were implanted into the bone defect through the posterior space. According to the severity of the bone defect, the position of the implanted bones was adjusted to be placed horizontally or vertically to meet the width of the vertebral space. Then, the distractor was released to check whether the implanted bones could be in close contact with adjacent vertebral bodies, enabling them to play a supporting role in anterior reconstruction. Autologous spinous bone and small bone fragments collected during the trimming were also used for posterior fusion. Afterward, appropriate compression was necessary to firm the bone grafting. After hemostasis and wound irrigation, streptomycin was locally performed in patients with tuberculosis, and vancomycin or sensitive antibiotics were locally used in patients with bacterial infection. Finally, a deep drainage catheter was placed on each side of the paravertebral region, and the incision was closed in layers.

### Postoperative management

All patients were required to rest in bed between 2 days and 3 weeks, and a lumbosacral orthosis was used to assistant ambulation. The orthosis was removed when imaging examination showed callus formation. Postoperative X-ray plain film or CT was taken to evaluate the fusion status. Six patients were received intravenous injection of antibiotics to treat pyogenic infection. The patient with tuberculotic spondylitis was treated with a standard isoniazid, rifampicin, ethambutol and pyrazinamide (HREZ) chemotherapy regimen for 12–18 months.

### Clinical assessment

Pain was assessed by the visual analog scale (VAS). The improvement of our surgical strategy on the patient’s daily activities was assessed by the ODI questionnaire. All patients were evaluated both before and after the operation at the final follow-up assessment. Postoperative X-ray plain film or CT was observed to evaluate the level of bony fusion at the operational sites. Postoperative complications were recorded.

### Statistical analysis

All analyses were performed using SPSS 25.0 software. Unless stated otherwise, values in the figures and text are presented as means ± standard deviation. Student’s *t* test (two tailed) was used for the comparation of preoperative and postoperative measurement data, including VAS and ODI scores. The chi-square test or Fisher’s precision probability test was used for count data, including sex and operational segment. A value of* p* < 0.05 was considered to be statistically significant.

## Results

### Patient population

All 7 patients were followed for at least 12 months and had detailed radiological and clinical data. The mean patient age was 63.57 (range 52–78) years, and the mean follow-up duration was 14.57 (range 12–18) months. Patients were diagnosed with chronic pyogenic spondylitis or tuberculosis based on clinical presentation, radiologic findings, and magnetic resonance imaging of osteomyelitis of the vertebral column based on clinical presentation, radiologic, microbial cultivation and tuberculin reaction. All 7 patients had infections with unsatisfactory conservative treatment because of back pain or leg pain. None of them showed paralysis. Each patient in our study underwent preoperative dynamic plain film X-ray, magnetic resonance imaging (MRI), and three-dimensional computed tomography (3D CT) scans. The background data for the included patients are shown in Table 1.

**Table 1:** Background data of study patients

Case No	Age (yr.)	Sex	Medical Problems	Symptoms	BMD	Fusion Levels	Follow Up Time (m)
1	63	F	Chronic Pyogenic Spondylitis	Back pain and leg pain	2.3	L4/L5L5/S1	19
2	52	F	Postoperative Pyogenic Spondylitis	Back pain	2.4	L5/S1	15
3	78	F	Tuberculotic Spondylitis	Back pain and leg pain	1.8	L5/S1	18
4	61	M	Postoperative Pyogenic Spondylitis	Back pain	2.1	L5/S1	12
5	57	M	Chronic Pyogenic Spondylitis	Back pain	1.6	L5/S1	12
6	69	F	Postoperative Pyogenic Spondylitis	Back pain	1.9	L5/S1	15
7	65	M	Chronic Pyogenic Spondylitis	Back pain and leg pain	2.2	L5/S1	15

*Yr.* year, *F* female, *M* male, *m* months

### Pain level

The pain level steadily and gradually decreased during the total follow-up period in all 7 patients. The preoperative VAS averaged 7.3 (range, 6–8). After the operation, it decreased significantly (P < 0.001) to an average of 2.6 (range, 2–3) (Table 2).

**Table 2:** Clinical data of patients

Case No	VAS			ODI (%)			Fusion time (m)
	Pre. op	Pos. op (3 d)	Pos. op (9 m)	Pre. op	Pos. op (3 d)	Pos. op (9 m)	
1	8	3	2	75	36	28	9
2	7	2	2	68	30	25	9
3	7	3	3	71	31	22	6
4	6	2	2	70	30	25	12
5	7	3	1	72	32	24	6
6	8	3	2	76	37	27	6
7	7	2	3	74	33	25	6
Mean	7.3 ± 1.3	2.6 ± 0.8	2.1 ± 0.8	73.8 ± 4.3	33.6 ± 2.9	29.2 ± 2.3	6.8 ± 1.3

*VAS* Visual analog scale, *ODI* Oswestry disability index

### Disability degree

The impact on the patient’s daily life was assessed by the ODI questionnaire. The ODI score demonstrated a steady and gradual increase throughout the total follow-up period. The average preoperative ODI score was 73.8 (range, 68–75). The score increased significantly (P < 0.05) to an average of postoperative 33.6 (range, 30–37) (Table 2).

### Complications

Two patients in our study experienced the complication of postoperative local pain within two months after the operation; their local pain was relieved by analgesics and disappeared 3 months after the operation. There was no fracture of the lateral sacral cortex during the operation of the S1 alar screw location and no posterior wound infections developed even in poor general condition. None of the patients developed neurovascular injury.

### Radiologic assessment of the fusion

**Fig. 2:**
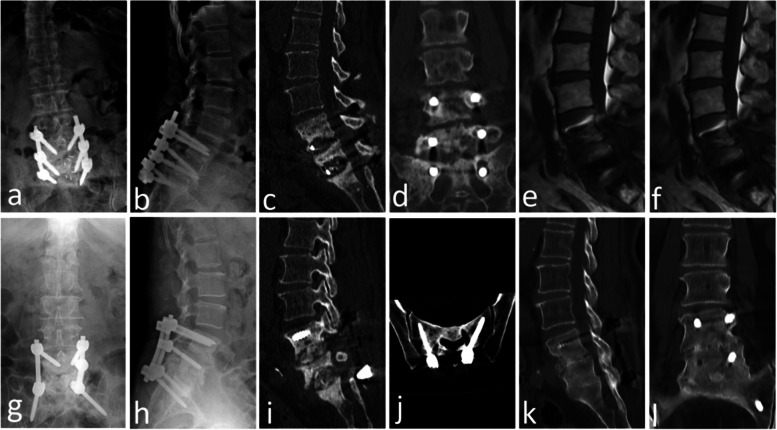
Case No. 1. A 63-year-old female underwent orthopedic revision surgery of lumbosacral fusion due to the complication of L4/5 and L5/S1 lumbosacral chronic pyogenic spondylodiscitis. S1 alar screws were used as substitutes for S1 pedicle screws to enhance biomechanical stability. (**a–f**) Preoperative X-ray, CT and MRI scans showed bone destruction with significant vertebral body loss at L4, L5 and S1. (**g, h**) X-ray images 3 days after the operation showed autologous grafting, and screws and rods were in good locations. (**i–j**) CT images 1 month after surgery showed the implanted bones and the trajectory of S1 alar screws in axial view. (**k–l**) CT images 19 months after surgery showed solid bone fusion from L4 and S1 without screws loosening

**Fig. 3:**
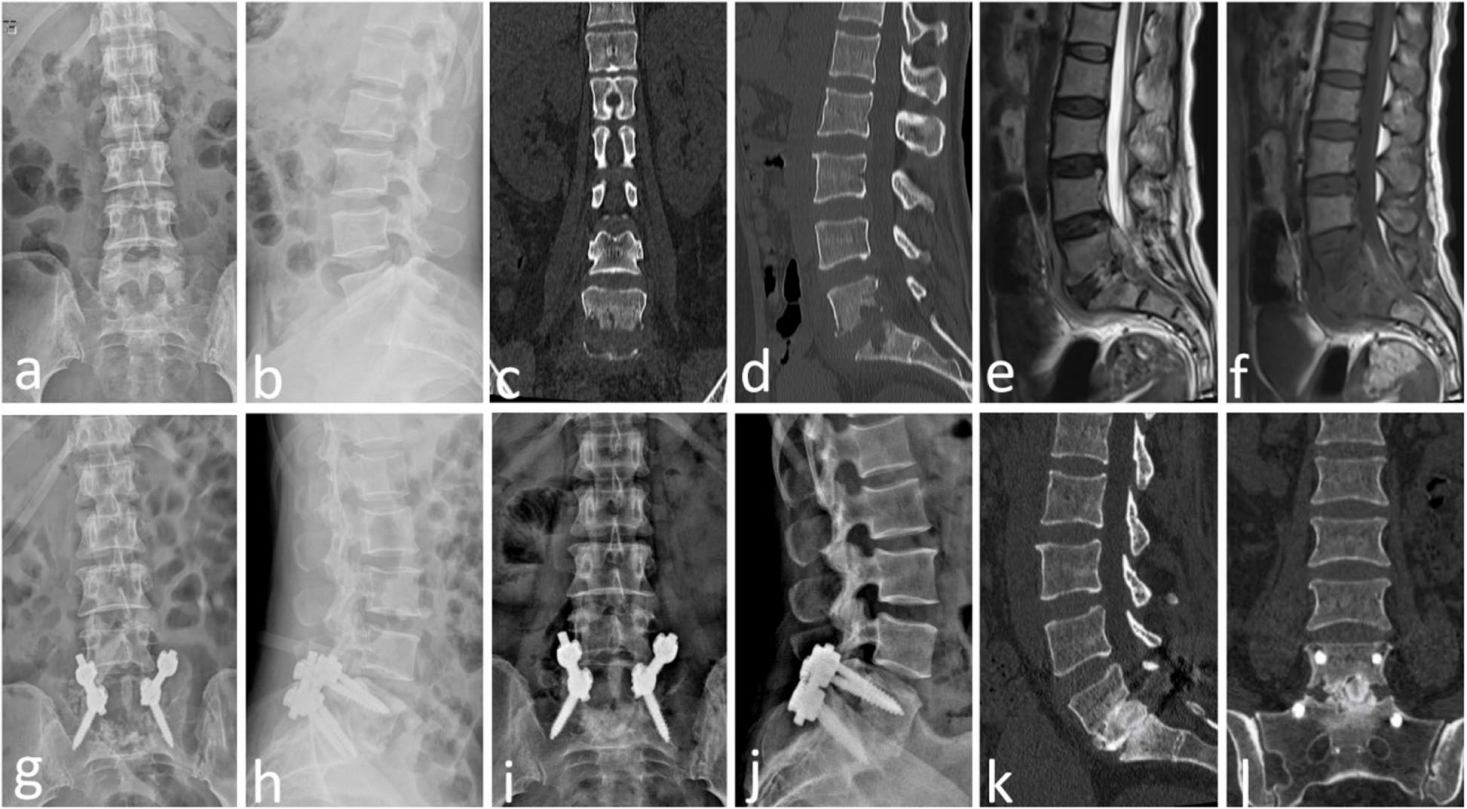
Case No. 2. A 52-year-old female underwent one-stage posterior surgery of L5-S1 due to a chronic E. coli infection. S1 alar screws were applied in single-segment lumbosacral fusion and fixation. (**a–f**) Preoperative X-ray, CT and MRI scans showed bone destruction with significant vertebral body loss at L5 and S1. (**g, h**) Postoperative X-ray images 4 days after the operation showed signs of posterior fusion and fixation of L5-S1 and autologous grafting, screws and rods in good location. (**i–j**) X-ray images 6 months after surgery showed partial bone fusion between L5 and S1. (**k–l**) CT images 12 months after surgery showed solid bone fusion between L5 and S1

On plain radiographs, radiologic evidence of stable bony fusion at the operational site was observed in all patients. Among them, 3 patients showed good fusion on CT scans, and 4 patients who refused CT examination reached the standard of fusion on X-ray, showing blurred intervertebral space and no screw loosening and breakage. Bony fusion was observed approximately 6.8 months after surgery (range: 6–12 months) (Table 2). All the patients showed solid fusion without pseudoarthrosis and two typical cases are shown in Fig. 2 and Fig. 3.

## Discussion

Currently, the surgical treatment strategies for spine infection should be made individually and carefully, as there are various options involving many aspects, such as appropriate approaches and surgical techniques, staging and instrumentation. According to reports, the addition of posterior instrumentation can provide faster rates of fusion and better deformity correction [15]; moreover, autogenous bone grafting implanted after dissection of the infectious tissues has been demonstrated to be effective and safe regardless of the causative organism [16]. In our study, we treated spinal infections with severe S1 vertebral body loss by using one-stage posterior instrumentation and interbody grafting with autogenous bone grafting after debridement, and satisfactory results of the correction of the deformity and fusion were achieved for all patients based on the follow-up assessments.

The key point in our study was to find appropriate fixation instruments for patients with severe S1 vertebral body loss as a lumbosacral infection. Currently, S2AI screws and iliac screws are the dominant fixation methods for the long-segment lumbosacral fixation and fusion because of the high fusion rate and low incidence of internal fixation-related complications [17]. However, in comparison, short-segment lumbosacral fixation is more inclined to end in the S1 segment to avoid additional local soft tissue destruction and the complicated operative technique required for accurate screw placement [10]. In previous reports, S1 alar screws have been applied commonly as an alternative instrument or a supplementary fixation for lumbosacral fusion [18]. Nevertheless, in our study, S1 alar screws were used as the main fixation screws of the sacrum to replace the S1 pedicle screws in short-segment fusion and fixation for lumbosacral joints.

The reason of the predominant use of S1 pedicle screws in lumbosacral fusion is the priority of biomechanics of the pedicle of the vertebral arch, and it has been reported that the BMD in the pedicle area is the highest comparing to that in other portions of the S1 vertebra [19]. Bone quality has been shown to have a significant effect on the strength of fixation in the sacrum and on the rates of early hardware failure [20]. According to the report, the BMD in the upper and lateral column of the sacral ala is the highest, except for that in the S1 pedicle area [14]. This suggests that the lateral mass of the sacral ala might be used as a potential site for anterior-lateral oriented S1 alar screws. The upper anterior-lateral trajectory was upper and lateral in the sacrum in the operation in our study to achieve greater bone strength.

In addition to the BMD discussed above, factors reported to influence the biomechanics of sacral screw fixations include screw lengths and diameters with corresponding osseous purchase and the bone cortex layers penetrated by the screw in the entry and exit point [21]. To acquire maximum biomechanical stability for the S1 alar screws, we designed and selected the unique entry point and the trajectory of the lateral sacral ala. The entry points and trajectories described in different studies are diverse [22]; however, the selection, the detail of the entry points and the trajectories are not discussed and clearly described. In our opinion, the entry points are important and are related to the trajectory length and orientation, which will eventually affect the biomechanical stability. The unique entry point of screws in our study was selected at the lateral inferior portion of the articular surface of L5 inferior articular process, where the strongest cortical bone of the sacral ala is concentrated. Wittenberg et al. [23] tested the biomechanical influence of different operation sites at level S1 and concluded that the pedicle screw inserted at level S1 through the S1 facet resulted in significantly higher pull-out forces than screws implemented by Harrington’s approach inserted to the entry point of the 10 mm lateral and 5 mm cephalad to the first dorsal sacral foramen. The axial pullout force of screws inserted at five sites was investigated by Zindrick et al. [24] to determine that the approach through the S1 facet was weaker only than an approach 45° laterally into the sacral ala and that the caudal site of screw insertion was associated with the most powerful strength. The cortical shell of the inferior S1 facet or around the pedicle of the sacrum used as the entry point in our study was thicker than the cortical shell of other area in the posterior sacrum.

Apart from the BMD and the entry point mentioned above, screw length by itself remains essential regarding pull-out strength [25]. McCord et al. [26] reported that the application of longer screws offers centralization of restraint regarding the lumbosacral pivot of rotation, which increases the stability of the instrumentation. The trajectory of the S1 alar screws described the first time in a previous study was directed 30° lateral and 30° distal [27]. However, the described S1 alar screw trajectory was aimed at cancellous bone and may not produce strong strength, and the fragile cancellous bone of the area may be related to the intraoperative complications of the fracture. The trajectory of the S1 alar screws should have an adequate lateral-inferior angle to obtain a satisfactory length based on the morphology of the sacral ala. Theoretically, penetration of the bilateral cortex could obtain sufficient strength. However, it may increase the risk of anterior nerve and vascular injury [28]. Anterior penetration carries the risk of damaging vital structures, such as the L5 nerve root, common iliac artery and vein, and midline sacral artery and vein [29]. As the screw would not puncture through the bone completely and should be oriented as lateral as possible, problems of screw penetration and injury to neural, vascular, and visceral structures could be avoided by the use of S1 alar screws in our study approach. These screws were directed as laterally as possible to obtain maximal length and to avoid injury to the internal iliac artery. However, to determine the optimal trajectory of the S1 alar screws, further study about radiological characteristics including maximal length, transverse angle and sagittal angle is needed.

In our study, satisfactory results of resolution of spinal infection and significant clinical recovery were achieved in all patients. The results of the VAS scores and the ODI score in our study showed that the postoperative outcomes were better than the preoperative results, indicating that the application of lumbosacral short-segment fixation with S1 alar screws was safe and effective. During the follow-up period, the pain and function scores were significantly improved compared with those before surgery. The fusion rate was up to 100% 12 months after surgery, and no loosening or failure of internal fixation was found during the follow-up period. Compared to the S2AI screws and iliac screws, the S1 alar screws have the advantage of easy connection to the L5 pedicle screws by the connecting rods and the reduction of soft tissue separation and resection, which can save a substantial amount of operation time and reduce blood loss considerably. In comparison to the anterior approach, the posterior approach using S1 alar screws can also reduce the potential risk of complications, such as vascular, lumbar plexus, colon, and pneumothorax injury [30]. Anterior penetration carries the risk of damaging vital structures, such as the L5 nerve root, common iliac artery and vein, and midline sacral artery and vein [29]. Similarly, Wu et al. [7] successfully treated 15 patients with lumbosacral tuberculosis with significant vertebral body loss by one-stage posterior surgical management using a structural autograft combined with a titanium mesh cage. Both titanium mesh and iliac bone can effectively construct anterior column defects in the posterior approach [31]. S1 alar screws would be indicated as a feasible alternative option when vertebral body loss at the level of the pedicle is more than 50% on preoperative 3D CT images.

In our study, surgery with the posterior approach made it difficult to correct the kyphosis caused by the anterior bone defects and soft tissue contractures in patients with spinal infection, and the sagittal balance in some patients seemed to be poor postoperatively in the lumbosacral segments. However, the patient's sagittal balance can be compensated by hyperextension of adjacent segments, and all patients had no significant low back pain for more than 3 months since the last follow-up. To solve this problem, a titanium mesh cage may provide strong support for the spinal anterior column, contributing to better correction of segmental kyphosis [32]. However, foreign materials such as titanium mesh cages may decrease antibiotic effectiveness and increase bacterial adherence. Furthermore, the short-segment surgery of one- or two-levels segments performed in our study was mainly to achieve effective spinal fusion with as little trauma as possible. Moreover, there is not enough operating space in the posterior approach to place a sufficiently large size of titanium cage for bone deficits caused by spine infection. There are also several shortcomings of S1 alar screws compared to pedicle screws, such as difficulty in screw placement, greater injury of the incision of the spinous process and articular process, and inferiority in BMD and screw length. Therefore, the S1 alar screws should not be used in patients with severe osteoporosis or in patients with long segment fixation.

There were still several limitations in this study. First and foremost, the sample size was small which may weaken the recommendation of this method for spine surgeons. Second, this was a retrospective, uncontrolled review of the clinical outcome of spinal surgery, and a randomized controlled trial is required in the future to verify the benefits and risks of our approach. Finally, further biomechanical and clinical studies should be performed to evaluate the characteristics of different trajectories in the sacral ala.

## Conclusions

Our research retrospectively evaluated the effectiveness of the one-stage posterior approach of short-segment fixation and fusion using S1 alar screws after debridement for lumbosacral infection with severe S1 vertebral body loss. We also detailed described the intraoperative use of the S1 alar screws as well as the unique entry point of the S1 alar screws. In conclusion, S1 alar screws could be an alternative to S1 pedicle screws for short-segment lumbosacral fixation and fusion with severe S1 vertebral body loss caused by spinal infection. This approach will provide satisfactory clinical outcomes.

## Data Availability

The datasets generated and analysed during the current study are not publicly available due to patient privacy protection but are available from the corresponding author on reasonable request.

## Abbreviations

VAS: Visual analogue scale
ODI: Oswestry disability index
BMD: Bone mineral density
S2AI: S2-alar-iliac
HREZ: Isoniazid rifampicin ethambutol and pyrazinamide
